# Serum protein biomarker profile distinguishes acetylcholine receptor antibody seropositive myasthenia gravis patients from healthy controls

**DOI:** 10.1016/j.isci.2024.110564

**Published:** 2024-07-23

**Authors:** Amol K. Bhandage, Viktorija Kenina, Yu-Fang Huang, Marija Roddate, Gundega Kauke, Arta Grosmane, Violeta Žukova, Niclas Eriksson, Katja Gabrysch, Tanel Punga, Anna Rostedt Punga

**Affiliations:** 1 Department of Medical Sciences, Clinical Neurophysiology, Uppsala University, Uppsala, Sweden; 2 Department of Neurology, Paul Stradinš Clinical University Hospital, Riga, Latvia; 3 Department of Neurology, Riga Stradinš University, Riga, Latvia; 4 Uppsala Clinical Research Center, Uppsala University, Uppsala, Sweden; 5 Department of Medical Biochemistry and Microbiology, Uppsala University, Uppsala, Sweden

**Keywords:** Neuroscience, Molecular neuroscience

## Abstract

There is an unmet need for objective disease-specific biomarkers in the heterogeneous autoimmune neuromuscular disorder myasthenia gravis (MG). This cross-sectional study identified a signature of 23 inflammatory serum proteins with proximity extension assay (PEA) that distinguishes acetylcholine receptor antibody seropositive (AChR+) MG patients from healthy controls (HCs). CCL28, TNFSF14, 4E-BP1, transforming growth factor alpha (TGF-α), and ST1A1 ranked top biomarkers. TGF-β1 and osteoprotegerin (OPG) differed between early- and late-onset MG, whereas CXCL10, TNFSF14, CCL11, interleukin-17C (IL-17C), and TGF-α differed significantly with immunosuppressive treatment. MG patients with moderate to high disease severity had lower uPA. Previously defined MG-associated microRNAs, miR-150-5p, miR-30e-5p, and miR-21-5p, correlated inversely with ST1A1 and TNFSF14. The presented inflammatory proteins that distinguish AChR+ MG are promising serum biomarkers for validation in prospective studies to allow for molecular signatures for patient subgroup stratification and monitoring of treatment response.

## Highlights

• An inflammatory protein biomarker signature defined AChR+ MG patients from healthy controls
• The top biomarkers of the 23 were CCL28, TNFSF14, 4E-BP1, TGF-α, and ST1A1
• MG subgroup-specific (onset, immunosuppression, severity) biomarkers were identified
• These findings allow prospective validation studies for patient subgroup stratification

## Graphical abstract

**Figure:**
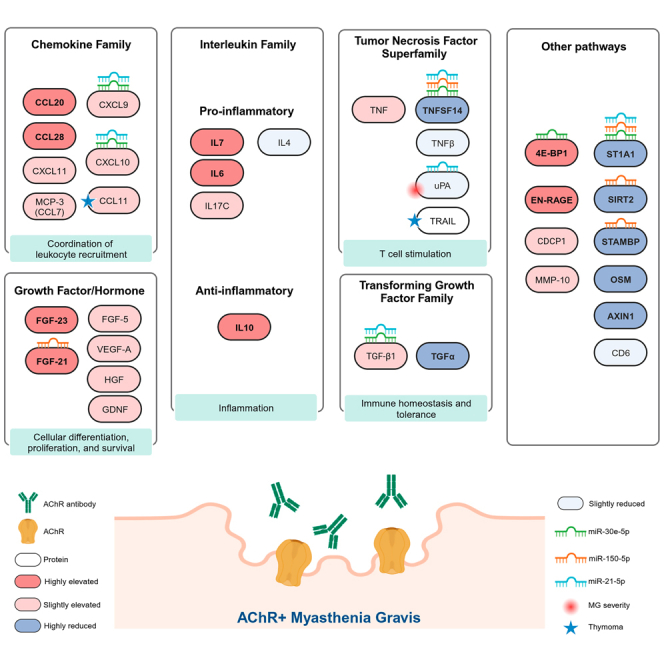

## Introduction

Myasthenia gravis (MG) is an autoimmune neuromuscular disorder where antibodies against postsynaptic receptors cause fluctuating skeletal muscle fatigue and weakness. MG is a highly heterogeneous disease with several subgroups based on serological status, clinical phenotype, age of onset, and thymic pathology association. The most common serological subtype of MG is acetylcholine receptor antibody seropositive (AChR+) MG, which can be subdivided into early-onset MG (EOMG) for patients with onset of disease at 19–50 years of age and late-onset MG (LOMG) with onset ≥50 years of age.^1^ AChR+ EOMG typically involves thymic follicular hyperplasia in young female patients, not observed in LOMG,^2^ where males are more commonly affected. There is a tremendous unmet need for objective MG-specific biomarkers in follow-up and prediction of disease activity over time, especially considering the emerging novel immunosuppressive treatments entering clinical trials in MG. Optimally, an MG biomarker would clearly distinguish MG patients from healthy individuals and reflect the underlying autoimmune pathophysiology. MG patients would benefit from treatment tailored to their disease subgroup and other possible disease biomarkers lacking today.^3^

A few studies have explored circulating microRNAs (miRNA) and various protein markers in MG to understand immune dysregulation. For example, elevated serum interleukins (ILs), IL-19, IL-20, IL-28, and IL-35,^4^ have been observed in MG patients, correlating with the disease severity. Another study found 11 inflammatory proteins to be elevated in MG sera compared to healthy controls, out of which matrix metalloproteinase 10 (MMP-10), transforming growth factor α (TGF-α), and extracellular newly identified receptor for advanced glycation end-products binding protein (EN-RAGE) were the most elevated.^5^ Further, elevated circulating miRNAs miR-150-5p, miR-30e-5p, and miR-21-5p have emerged as potential serum biomarkers for AChR+ MG, with reduced levels after immunosuppression and thymectomy.^6^^,^^7^^,^^8^^,^^9^

The main objective of this study was to define a serum protein profile that separates AChR+ MG patients from healthy controls (HCs) for further validation as MG biomarkers. Secondary objectives were to assess whether the inflammatory proteins were altered in EOMG versus LOMG and patients with and without immunosuppressive therapy. Finally, we correlated the serum protein levels with the levels of circulating microRNAs miR-150-5p, miR-30e-5p, and miR-21-5p levels, previously found elevated in AChR+ MG.

## Results

### Distinct inflammatory protein biomarker profile in AChR+ MG

**Figure 1:**
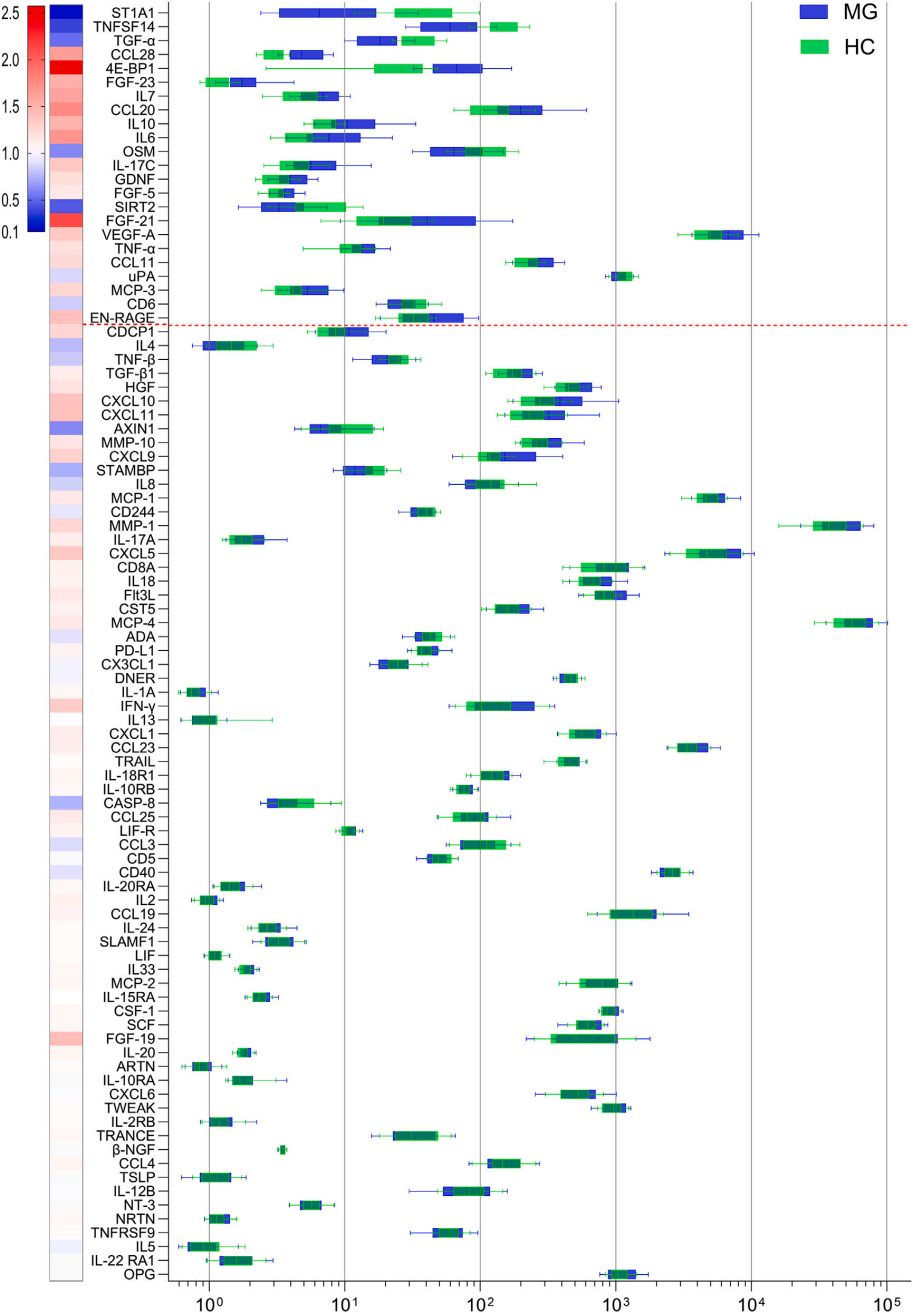
Serum protein biomarker profile in AChR+ MG Screening of Olink Target 96 Inflammation Panel with 92 proteins in serum samples from 98 MG patients (blue) and 77 HCs (green). Data are represented as the median 2^NPX^ values and 10^th^ to 90^th^ percentile range, sorted by *p* value from the logistic regression analysis, Model 1, with age and sex correction. The proteins above red lines indicate differential expression in MG and HC groups with the Bonferroni correction significance cutoff value *p* < 0.0006. The heatmap on the left side indicates a fold change in the median value from MG patients to the median value from HC for each corresponding protein.

This cohort study included serum samples from 98 AChR+ MG patients [(38 males (38.8%), median age: 61 years (IQR 44.2–72.0 years; Table 1)] and 77 matched HCs [(31 males (40.3%), median age: 55 years (IQR 41.0–64.0 years)]. MG disease duration ranged from 0 to 50 years, MG activities of daily living (MG-ADL) scores ranged from 0 to 16 points, and myasthenia gravis composite (MGC) score ranged from 0 to 21 points (Table S1). Serum levels of 92 inflammatory proteins (Table S2) were quantified. To identify biomarkers that would separate important MG subgroups, we analyzed 46 EOMG and 52 LOMG patients (Table 1). These patients included 28 without immunosuppressant treatment and 70 with immunosuppressant treatment. Protein levels were correlated with MG severity, thymectomy status (including thymoma or hyperplasia), clinical characteristics, and levels of miR-150-5p, miR-30e-5p, and miR-21-5p in MG and HC sera. Logistic regression, performed on NPX values with the MG group as the outcome, revealed statistical significance with Bonferroni corrected *p* value (*p* < 0.0006) for 27 proteins in model 0 and 23 proteins in model 1 (with sex and age correction; Table S3). Sixteen proteins were significantly higher, and seven were significantly lower in MG patient sera (*p* < 0.0006; Figure 1). CCL28, FGF-23, FGF-5, TGF-α, TNFSF14, and uPA had the highest difference between MG and HC.

**Table 1:** Clinical characteristics of the MG patients included in the study

	All MG	EOMG	LOMG
Total patients	98	46	52

**Sex**			

F	60 (61.2%)	37 (80.4%)	23 (44.2%)
M	38 (38.8%)	9 (19.6%)	29 (55.8%)
Mean age (y)	61.0 (44.2–72.0)	43.5 (34.0–51.8)	72.0 (65.8–77.0)
Disease duration (y)^b^	5.8 (2.0–11.0)	7.5 (3.4–13.6)	4.0 (2.0–8.8)
MG-ADL	2 (0–5)	3 (0–6)	1 (0–4)
MGC	4 (0–9.75)	5 (0–11)	2 (0–7)
Thymectomy	24 (24.5%)	19 (41.3%)	5 (9.6%)
Thymoma	14	10	4
Hyperplasia	6	5	1

**Immunosuppressive treatments**			

Yes	70 (71.4%)	34 (73.9%)	36 (69.2%)
No	28 (28.6%)	12 (26.1%)	16 (30.8%)

**Current treatment**^a^			

AChEI (only)	20 (20.4%)	9 (19.6%)	11 (21.2%)
Prednisone	53	30	23
Azathioprine	29	15	14
Tacrolimus	1	1	0
Rituximab	3	2	1
IvIg	2	2	0
Mycophenolate mofetil	5	4	1
Methotrexate	8	4	4

Age, disease duration, and MG-ADL are presented as median with interquartile range. *Abbreviations:* F, female; M, male; y, years; EOMG, early-onset myasthenia gravis; LOMG, late-onset myasthenia gravis; MG-ADL, myasthenia gravis activities of daily living; MGC, Myasthenia Gravis Composite score; AChEI, acetylcholinesterase inhibitors; IvIg; intravenous immunoglobulins.

a The number of detailed immunosuppressive treatments does not add up to 100% since several patients have a combination of many immunosuppressive agents.

b EOMG and LOMG significantly differed in disease duration; unpaired t test; *p* = 0.0007.

**Figure 2:**
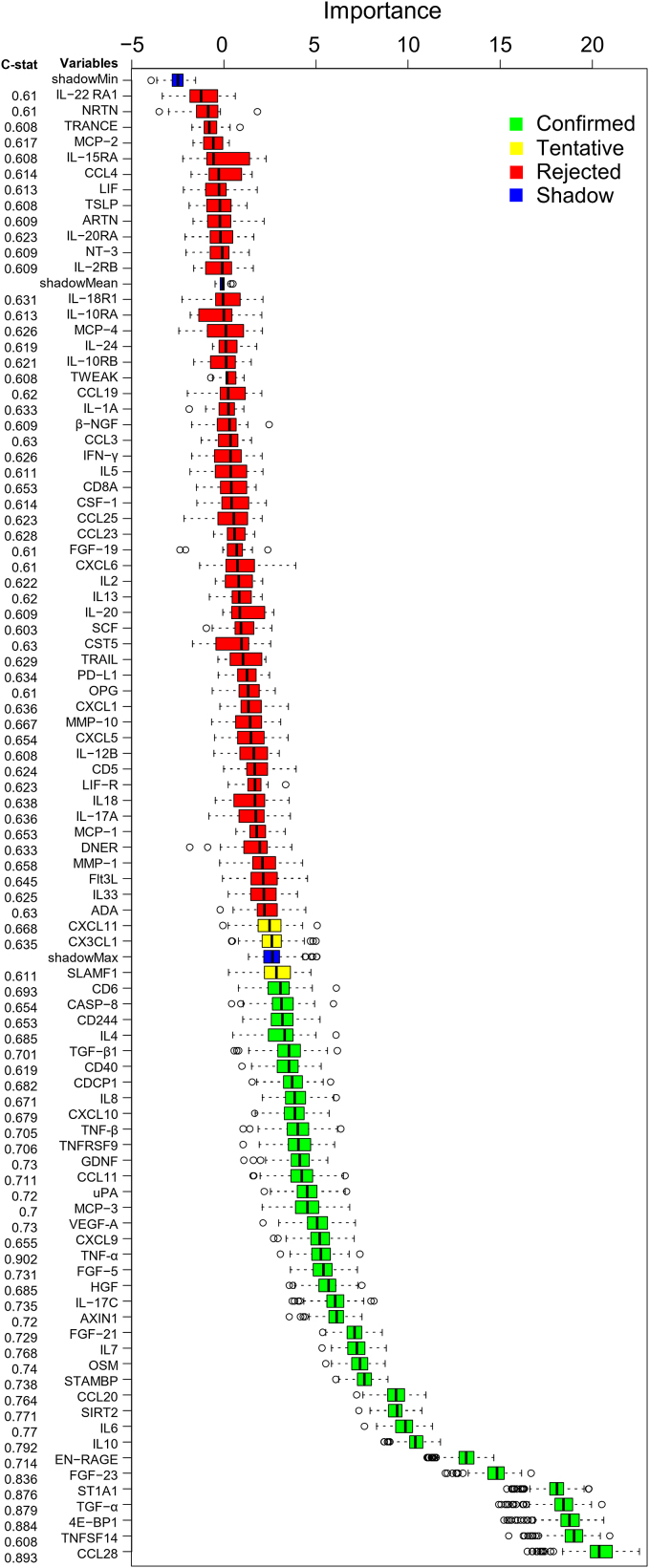
Boruta algorithm analysis plotted as importance values for all 92 proteins sorted with increasing median importance values, accompanied by the c-statistic value indicating the area under the curve in ROC curves for all proteins. Data are represented as the median and 5^th^ to 95^th^ percentile range.

**Figure 3:**
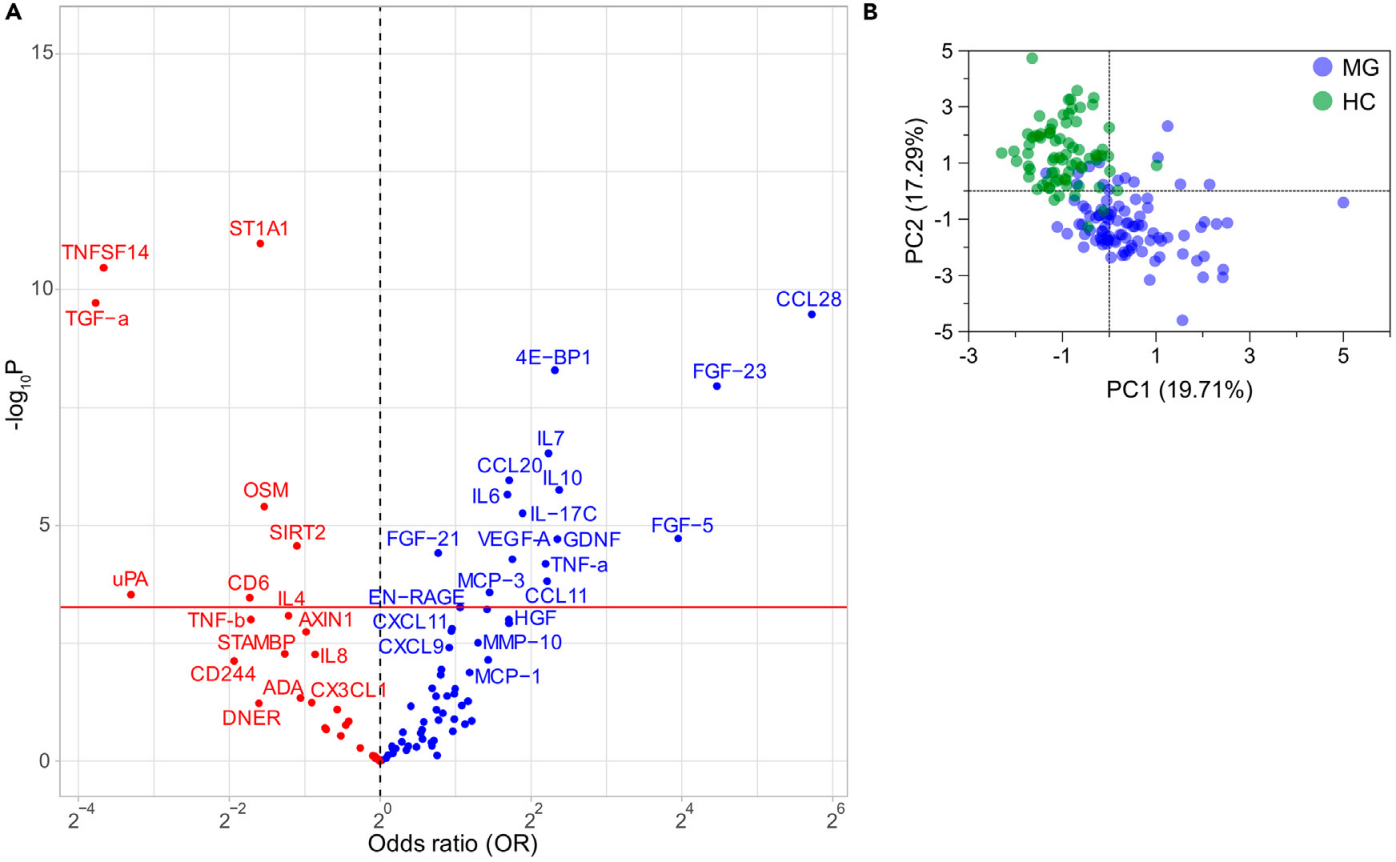
Separation of protein biomarkers visualized as Volcano and PCA plots (A) Volcano plot, with *p* values and odds ratio (OR), for differential expression in MG against HC. The proteins above red lines indicate the Bonferroni correction significance cutoff value *p* < 0.0006. (B) PCA plot of top 20 biomarkers with highest median importance values in Boruta algorithm analyses indicative of separation between MG patients and HC groups for each individual.

Boruta algorithm confirmed proteins as MG biomarkers and ranked them by importance value, with the most important biomarkers being CCL-28, TNFSF14, 4E-BP1, TGF-α, ST1A1, FGF-23, EN-RAGE, IL-10, IL-6, and SIRT2 (Figure 2). A Volcano plot shows the distribution of all proteins between all patients and HCs (Figure 3A), whereas a PCA plot of the top 20 proteins visualizes the separation of each patient and HC (Figure 3B).

Notably, the previously identified elevated levels of MMP-10, TGF-α, EN-RAGE, IL-6, IL-17C, and IL-10^5^ in mixed MG patients were confirmed elevated in AChR+ MG patient sera (Table S3).

### Correlation between protein biomarkers and microRNA levels

Spearman correlation matrix of 92-X-92 proteins revealed 242 common positive correlations in both groups (Figure S1A; *p* ≤ 0.01), whereas 245 and 481 separate positive correlations were observed in MG and HC groups, respectively (Figures S1B and S1C; *p* ≤ 0.01). Among MG patients, age had a moderate positive correlation with OPG and a weak positive correlation with CXCL9, CDCP1, IL-8, CCL11, and FGF-5. Further, age had a weak negative correlation with TGF-β1 and TRANCE levels (Table 2). Disease duration had a weak positive correlation with FGF-23, MCP-1, and IL-13 and a weak negative with CD8A and IL-5 levels. MG-ADL and MGC both had a weak negative correlation with uPA and Flt3L.

**Table 2:** Correlation analysis

	Spearman r	95% CI	*p* value	# XY pairs
**Age (years) vs.**				

OPG	0.4499	0.2707 to 0.5990	<0.0001	98
TGF-β1	−0.2949	−0.4707 to −0.09659	0.0032	98
TRANCE	−0.3004	−0.4838 to −0.1134	0.0019	98
CXCL9	0.2971	0.09897 to 0.4726	0.003	98
CDCP1	0.2772	0.07751 to 0.4556	0.0057	98
IL-8	0.3068	0.1205 to 0.4893	0.0022	98
CCL11	0.3053	0.1079 to 0.4795	0.0022	98
FGF-5	0.2977	0.09960 to 0.4731	0.0029	98

**Disease duration (years) vs.**				

FGF-23	0.3038	0.06302 to 0.4440	0.0042	98
MCP-1	0.2651	0.06442 to 0.4451	0.0083	98
IL-13	0.3007	0.08125 to 0.4586	0.004	98
CD8A	−0.2821	−0.4683 to −0.09358	0.0075	98
IL-5	−0.2815	−0.4737 to −0.1005	0.0064	98

**MG-ADL vs.**				

uPA	−0.3208	−0.4757 to −0.1030	0.0016	98
Flt3L	−0.2654	−0.4454 to −0.06262	0.0089	98

**MGC vs.**				

uPA	−0.2813	−0.4608 to −0.07967	0.0055	96
Flt3L	−0.2731	−0.4538 to −0.07085	0.0071	96

**miR-30e-5p vs.**				

ST1A1	−0.4624	−0,6215 to −0,2667	<0.0001	82
TNFSF14	−0.3584	−0,5385 to −0,1469	0.0009	82
TGF-β1	−0.2904	−0,4823 to −0,07182	0.0081	82
CXCL9	0.3171	0.06822 to 0.4795	0.0043	82
CXCL10	0.3281	0.08026 to 0.4888	0.0026	82

**miR-150-5p vs.**				

ST1A1	−0.6035	−0.7359 to −0.4522	<0.0001	82
TNFSF14	−0.4644	−0.6308 to −0.2810	<0.0001	82
SIRT2	−0.4224	−0.5900 to −0.2199	<0.0001	82
CXCL1	−0.3557	−0.5355 to −0.1428	0.0011	82
STAMBP	−0.3524	−0.5337 to −0.1403	0.0012	82
FGF-21	0.3345	0.1314 to 0.5272	0.0028	82
β-NGF	−0.2896	−0.4817 to −0.07095	0.0083	82
CXCL6	−0.3083	−0.4973 to −0.09137	0.0048	82

**miR-21-5p vs.**				

ST1A1	−0.3503	−0.5319 to −0.1378	0.0013	82
TNFSF14	−0.4043	−0.5756 to −0.1991	0.0002	82
CXCL9	0.4406	0.2411 to 0.6044	<0.0001	82
uPA	0.3798	0.1711 to 0.5559	0.0004	82
OPG	0.4372	0.2371 to 0.6017	<0.0001	82
CXCL6	−0.3216	−0.5083 to −0.1060	0.0032	82
CSF-1	0.3367	0.1227 to 0.5208	0.002	82
IL-15RA	0.3109	0.09426 to 0.4995	0.0045	82
CXCL1	−0.2869	−0.4795 to −0.06810	0.009	82
Flt3L	0.3236	0.1082 to 0.5100	0.003	82
CXCL10	0.3211	0.1055 to 0.5079	0.0033	82
TGF-β1	−0.2867	−0.4792 to −0.06781	0.009	82
CDCP1	0.353	0.1409 to 0.5341	0.0011	82
OSM	−0.3157	−0.5035 to −0.09953	0.0039	82
PD-L1	0.3054	0.08817 to 0.4949	0.0053	82

Statistically significant correlations between demographic characteristics or miRNA expression in the serum of MG patients and the 92 assayed inflammatory protein markers.

Next, we found that serum miR-150-5p, miR-30e-5p, and miR-21-5p levels correlated with 8, 5, and 15 protein markers in the MG group and with 0, 1, and 2 protein markers in HC groups, respectively (Table 2). ST1A1 and TNFSF14 correlated with all three miRNAs. ST1A1 had a strong negative correlation with miR-150-5p and miR-30e-5p, and TNFSF14 had a strong negative correlation with miR-150-5p and miR-21-5p. TGF-β1, CXCL1, CXCL6, CXCL9, and CXCL10 correlated with at least two miRNAs (Table 2).

According to the target scan analysis, miR-21-5p has a single binding site at the 3′UTR of TGF-β1; no other binding sites were found on the dysregulated proteins for the other miRNAs.

### Associations of protein biomarkers with subgroups and clinical characteristics

**Figure 4:**
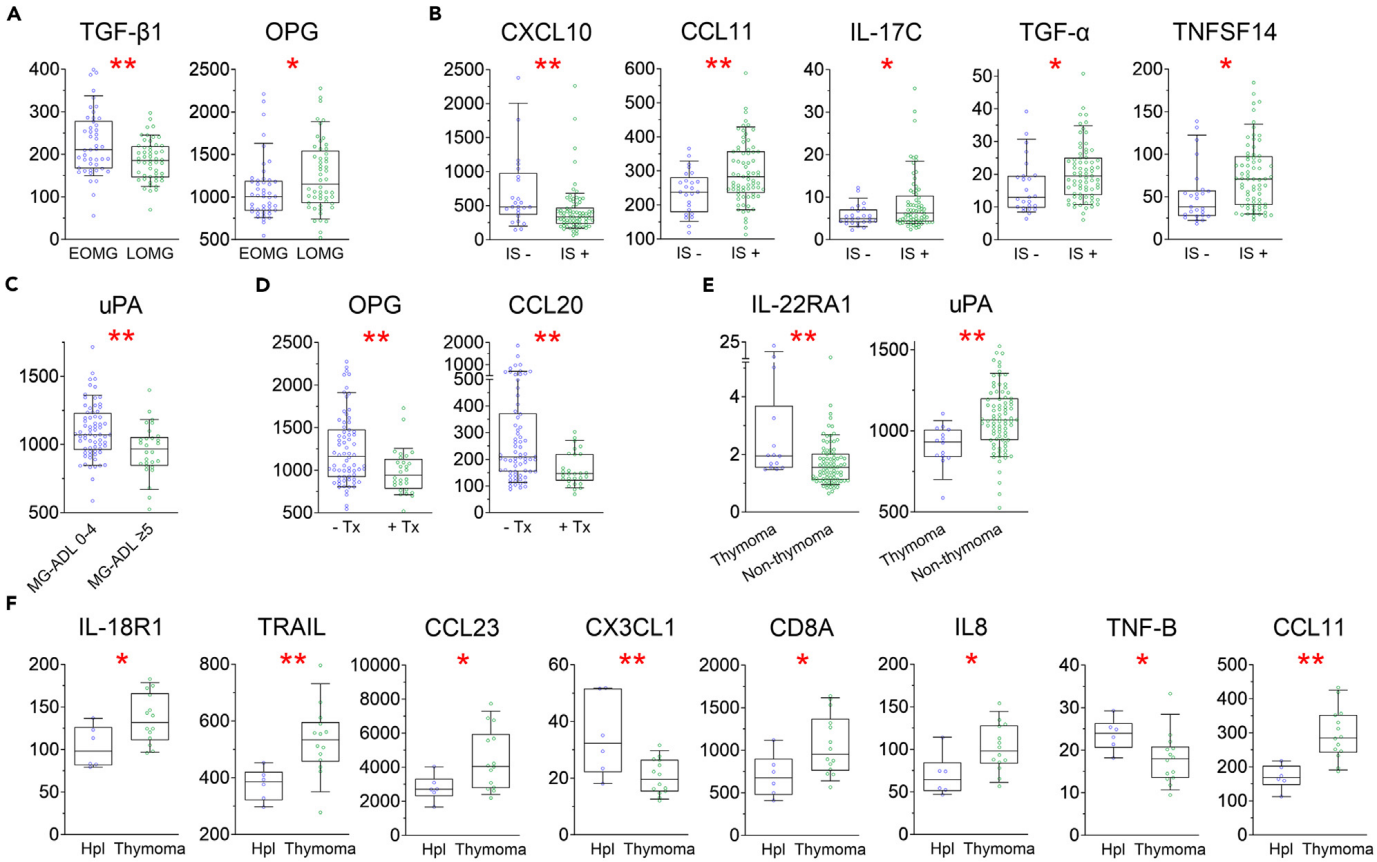
Identification of protein biomarkers in MG subgroups Data are 2^NPX^ values of the proteins with significantly different levels in MG sub-groups, represented as the median and 10^th^ to 90^th^ percentile range. (A) EOMG and LOMG. (B) MG patients without and with immunosuppressants (IS). (C) MG patients with low (0–4) and moderate to high (≥5) severity on MG-ADL. MG patients with and without (D) thymectomy (Tx) or (E) thymoma. (F) MG patients with hyperplasia (Hpl) and thymoma. ∗*p* ≤ 0.05, ∗∗*p* ≤ 0.01. Related Extended Data Table S4 delineates logistic regression analysis.

Our second aim was to identify protein biomarkers that could separate EOMG and LOMG and MG patients with and without immunosuppressants. Logistic regression revealed significant differences for TGF-β1 and OPG between EOMG and LOMG (Figure 4A; Table S4). Further, CXCL10, TNFSF14, CCL11, IL-17C, and TGF-α differed significantly between MG patients with and without immunosuppressants (Figure 4B).

We next assessed exploratory correlations based on clinical characteristics in MG patients. MG patients with moderate to high disease severity had lower uPA levels (Figure 4C). MG patients who underwent thymectomy had lower levels of OPG and CCL20 (Figure 4D). Thymoma patients had higher levels of IL-22RA1 and lower levels of uPA (Figure 4E). Patients with hyperplasia and thymoma differed in several proteins, including IL-18R1, TRAIL, CCL23, CX3CL1, CD8A, IL-8, TNF-β, and CCL11 (Figure 4F).

## Discussion

**Figure 5:**
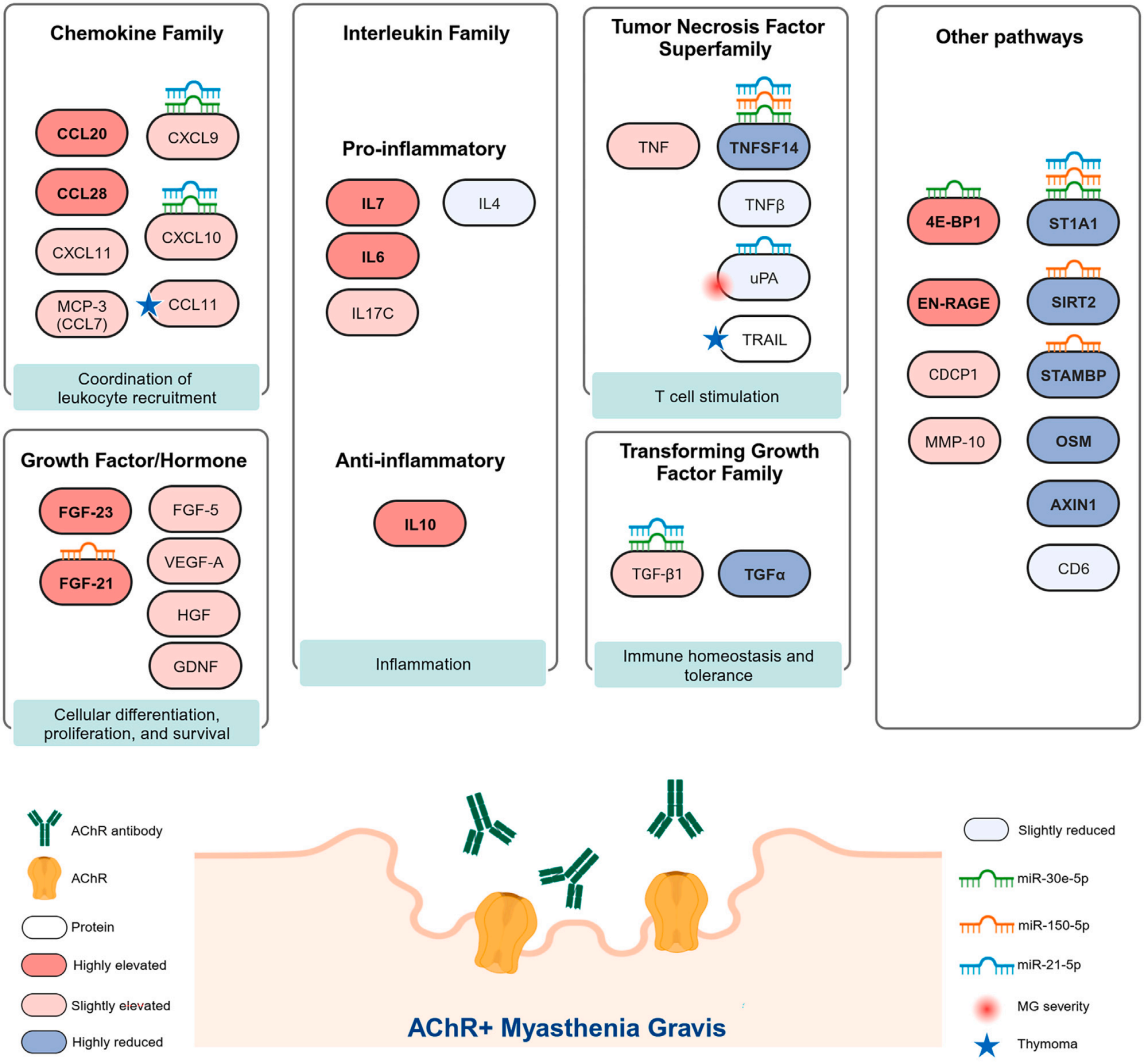
Summary of serum protein biomarker changes in AChR+ MG Elevated proteins in MG are delineated in different shades of red, and reduced proteins are delineated in different shades of blue. Proteins in which levels correlate with microRNAs miR-150-5p, miR-21-5p, and miR-30e-5p are marked with their respective symbols. Symbols also mark associations between proteins and MG severity as well as thymoma.

Despite the steady improvement in treating autoimmune inflammatory neuromuscular diseases, including MG, essential pathophysiological processes underlying the chronic autoimmune response and muscle fatigue in AChR+ MG remain unknown. Recognizing specific inflammatory protein biomarkers relating to distinct immune pathways confers additional insights into the underlying pathophysiology while offering the possibility to use molecular phenotypes in deciding what treatment to choose instead of relying on experience-based subgroups related to age, sex, etc.^1^^,^^10^ Therefore, developing objective and reliable MG biomarkers in disease diagnosis, follow-up, and predicting disease activity is needed for clinical follow-up and clinical trials and is considered one of the top priority areas in MG research. Although AChR antibodies are an essential diagnostic tool, there is insufficient evidence to depict AChR antibody levels as longitudinal biomarkers for disease activity in MG.^11^ Here, we present a distinct serum profile of inflammatory protein biomarkers that can separate AChR+ MG patients from HCs (Figure 5) and is representative of a broad activation of the immune system, with the recruitment of both the innate and acquired immune responses.

The top 1 biomarker candidate, C-C chemokine ligand 28 (CCL28), which recruits primarily IgA-producing B cells, and CCL20 (11^th^ top biomarker), which recruits dendritic and T cells to the sites of inflammation in mucosal tissues, were elevated by about 75% in MG patients. CCL28 has previously not been linked to MG; however, based on its immune cell recruiting properties, it could be important for MG pathogenesis. CCL28 is elevated in mucosal tissues in inflammatory bowel diseases and synovial tissues in rheumatoid arthritis (RA).^12^ CXCL9, CXCL10, and CXCL11 are other upregulated chemokines, typically produced in response to interferon-gamma (IFN-γ). CXCL10 and its receptor, CXCR3, are overexpressed in the thymus, muscles, and CD4^+^ T cells of AChR+ MG patients.^13^

The 2^nd^ top biomarker, TNFSF14 (also known as LIGHT), promotes the differentiation of various inflammatory cells and IL-6 production, inhibits T cell activation, and reduces Th1 chemokine expression in other autoimmune disorders.^14^ LIGHT gene polymorphism is associated with lower TNFSF14 serum levels and an increased risk of multiple sclerosis (MS).^15^ Serum TNFSF14 levels are higher in MS relapse and reduced upon immunosuppressive treatment with natalizumab.^15^ In contrast, TNFSF14 levels were lower in our MG cohort, especially in patients without immunosuppression. In addition to TNFSF14, several other members of the tumor necrosis factor (TNF) superfamily of cytokines, including TNF-α, TNF-β, uPA, and the TGF family, TGF-β1 and TGF-α, were dysregulated in MG (Figure S1), indicating interconnections of the mitogen-activated protein kinase (MAPK) and nuclear factor κB (NF-κB) pathway, in which uPA is one of the end products. Our data, together with previous data on systemic TNF-producing CD103^+^ Th cells representing a disease-specific signature population in MG,^16^ emphasizes the role of the TNF superfamily in the pathogenesis of MG.

The 3^rd^ most important MG-biomarker, eukaryotic translation initiation factor 4E-binding protein 1 (4E-BP1), correlated inversely with TNFSF14. The mTORC1-4E-BP1-eIF4E axis controls the translation of many mRNAs that encode central immune-regulatory proteins, including IL-4, which is expressed by CD4^+^ T helper cells and regulates B cell expansion and was reduced in MG patients, in line with a previous report.^17^ Elevated 4E-BP1 levels in MG could imply dysregulation of the mTORC1–4E-BP–eIF4E axis, resulting in T cell dysfunction and autoimmunity. ST1A1 levels were markedly reduced, and ST1A1 was the 4^th^ most important biomarker separating MG from HC. Intriguingly, this protein was significantly associated with the proteins CASP-8, AXIN1, SIRT2, and STAMBP, indicating their interaction in T cell regulating pathways.^18^

Of the previously identified elevated proteins in MG sera,^5^ high levels of five proteins, TGF-α, EN-RAGE, IL-6, IL-17C, and IL-10, were importantly replicable and confirmed. Elevated IL-6 and IL-17C levels in MG patients are most likely due to increased Th1 and Th17 cells, also observed in the sera of RA patients,^19^ and dampened T regulatory cell (Treg) suppressive capabilities. In our MG cohort, TGF-α levels were lower than in HCs. Despite no previous link between TGF-α and autoimmune disorders in the literature, this protein can potentially promote the generation of Tregs and interact with other cytokines.

The circulating microRNAs miR-150-5p, miR-30e-5p, and miR-21-5p are potential serum biomarkers in AChR+ MG.^9^ Lower miR-150-5p levels in CD4^+^ T cells^20^ and higher levels in the serum of AChR+ MG^7^ patients suggest the release of miR-150-5p from activated peripheral CD4^+^ T cells. Our findings of several correlations in MG patients between these miRNAs and dysregulated proteins, but not in HCs, indicate that miR-150-5p, miR-30e-5p, and miR-21-5p may indirectly regulate these mRNAs and protein levels in MG. In particular, TNFSF14 and ST1A1 had an inverse correlation with all three miRNAs. The only direct binding site found was for miR-21-5p on TGF-β1.

One of the main issues in MG is that the disease is highly heterogeneous and that the patients belong to different subgroups based on the age of onset, serological status, and treatment status, in addition to presenting with wide clinical heterogeneity, e.g., symptom distribution with variability in skeletal muscle involvement, disease grade, thymectomy status, thymic histology, and disease duration. However, we tried to keep the disease subgroups homogeneous and corrected for age and sex. Our second aim was to determine potential differences in protein levels between the most commonly used subgroups. EOMG and LOMG are often considered different disease entities due to the link between EOMG and thymus hyperplasia in young females. On the other hand, patients with LOMG more often have a normal thymus, which is more common in men than women.^21^ Nevertheless, we could only detect rather modest differences in the two proteins OPG and TGF-β1. Higher levels of TGF-β1, which regulates responses of CD4^+^ T cells and B cells,^22^ were seen in EOMG. Further, OPG levels were higher in LOMG, possibly due to increased bone mass with age, as observed in previous studies.^23^ Our data thus support another study indicating that there may be an overlap between EOMG and LOMG, especially between 40 and 60 years of age.^24^ Another factor for differences between EOMG and LOMG could be sex due to the predominance of female patients in the EOMG subgroup. Immunosuppression is used in most MG patients; however, the choice of immunosuppressant medications and mechanisms of action differ. Despite the several immunosuppressants used, where the majority received corticosteroids and azathioprine to fewer patients receiving methotrexate, rituximab, etc., we only found differential levels of five proteins, CXCL10, TNFSF14, CCL11, IL-17C, and TGF-α, between MG patients with and without immunosuppressants. We did not find it feasible to perform any analyses of immunosuppression type subgroups within this study. Regarding clinical severity, lower levels of uPA separated MG patients with moderate to severe MG severity from those having none to mild symptoms. Further, several proteins were found to differ in patients with and without thymus hyperplasia or thymoma. This indicates the importance of recognizing these thymus subgroups in MG for the immunological pathways, in line with previous studies.^2^ Nevertheless, we had a relatively high percentage of thymoma and a lower group with thymus hyperplasia in our cohort, which is in line with prior studies showing that almost 22% of Latvian MG patients have a thymoma and only 2% have thymus hyperplasia.^25^ This difference may be attributed to the local diagnostic approach in Latvia, wherein radiologists often only report thymic changes when thymoma is apparent, potentially leading to underreporting more subtle thymic alterations.

The strength of our study is that we have a relatively large homogeneous group of AChR+ MG patients from two different European countries, considering the rarity of the disease compared to other neuroimmunological disorders, such as MS. We were able to define highly significant protein profiles as biomarkers that separated MG from HCs and that also separated subgroups, which could indicate important differences reflecting MG immunopathogenesis. A prospective follow-up multi-center study before and after treatment, and comparison to other neuroimmune diagnoses, would be required to validate the sensitivity, specificity, and usefulness of the defined inflammatory protein profile in the follow-up of treatment response and predict the disease course in individual MG patients.

In conclusion, this study defined a profile of inflammatory serum protein biomarkers that separates the largest group of AChR+ MG patients from HCs (Figure 5). The data especially indicate the importance of the proteins CCL-28, TNFSF14, 4E-BP1, TGF-α, ST1A1, FGF-23, EN-RAGE, IL-10, IL-6, SIRT2, and CCL20 in the pathogenesis and the chronic autoimmune response of AChR+ MG. These findings expand our knowledge of the immunological pathways associated with AChR+ MG. They will allow prospective validation studies of promising serum biomarkers in MG to monitor and predict treatment response and select patients for novel treatment options. Ultimately, this will enable the stratification of MG patients into various subsets based on molecular signatures, guiding personalized treatment in this chronic autoimmune neuromuscular disorder.

### Limitations of the study

The study’s main limitation was that most patients were on immunosuppressive treatment, which can influence the levels of inflammatory serum proteins. Nevertheless, an equal number of EOMG and LOMG patients were on immunosuppressive treatment. Next, due to its cross-sectional design, this study cannot prove a causal relationship between the differentially expressed protein biomarkers and pathophysiological pathways in MG. It should also be noted that protein rankings based on *p* values from logistic regression analyses or importance values from Random Forest analyses are uncertain, and an independent study of the same size would probably replicate associations passing thresholds for multiple tests but not the ranking compared to other proteins.

## STAR★Methods

### Key resources table

**Table:** 

REAGENT or RESOURCE	SOURCE	IDENTIFIER
**Biological samples**		

Serum samples from MG patients	Uppsala University Hospital, Uppsala, Sweden. Paul Stradinš Clinical University Hospital, Riga, Latvia.	N/A
Serum samples from healthy controls	Uppsala University Hospital	N/A

**Critical commercial assays**		

Olink Target 96 Inflammation panel	Olink Proteomics AB	95302
miRNeasy® Serum/Plasma Advanced kit	QIAGEN	217204
RNA Spike-in kit for RT	QIAGEN	339390
MS2 bacteriophage RNA	Roche	
miRCURY® LNA® RT kit	QIAGEN	339340
miRCURY® LNA® miRNA custom PCR Panel	QIAGEN	YCA42100 (customized)
miRCURY® LNA® SYBR® Green PCR kit	QIAGEN	339345

**Software and algorithms**		

QuantStudio Real-Time PCR software v1.7.2	Applied Biosystem	N/A
GraphPad Prism 10.1	GraphPad	N/A
R version 4.2.3	R Core team	N/A

### Resource availability

#### Lead contact

Further information and requests should be directed to and will be fulfilled by the lead contact, Prof. Anna Rostedt Punga, Uppsala University, Uppsala (anna.rostedt.punga@uu.se).

#### Materials availability

This study did not generate new unique reagents.

#### Data and code availability

• No original code has been generated in this study.
• The data and materials supporting the conclusions of this article are included in this published article (and its supplemental information files).
• The primary Olink data and related sample metadata are available from the lead contact upon reasonable request. Still, individual-level data can only be released under a suitable data-sharing agreement due to informed consent restrictions.

### Experimental model and study participant details

#### Study design, power calculations, and patient cohorts

The hypothesis was that MG patients have a defined inflammatory protein profile in sera compared to HCs. Effect estimates determined with linear regression analysis of inflammatory protein levels (MMP-10 with the highest p-value and IL-17A with the lowest p-value, from our previous publication^5^) between MG patient and healthy control (HC) groups were used as reference points in the t-test power section. To obtain a power of 0.8 with the Bonferroni adjustment for multiple tests, at least 50 MG patients and 50 healthy controls had to be included. To increase the power and to control for the potential effect of subgroups, such as onset and immunosuppressive therapy, we included 98 MG patients [46 with EOMG (onset at 19-50 years of age), and 52 with LOMG (onset ≥ 50 years of age)]. In the HC group, we included 77 age- and sex-matched healthy blood donors.

MG patient sera were obtained from AChR+ MG patients at the Department of Neurology, Uppsala University Hospital, and the Department of Neurology, Paul Stradiņš University Hospital. All patients had an ICD-10 diagnosis of G70.0, a positive AChR antibody titer on radio immune assay, and abnormal neuromuscular transmission by repetitive nerve stimulation (RNS) or single fiber electromyography (SFEMG). Matched HC sera were obtained from an existing biobank of 200 serum samples (100 males and 100 females) collected in 2021 at the transfusion unit of Uppsala University Hospital.

Clinical records of all MG patients were reviewed to assess sex, age at MG onset, timing from symptoms onset to diagnosis, clinical subgroup and severity, electrophysiological data (RNS/SFEMG), ongoing and previous treatments, and disease status at follow-up. All patients were diagnosed and evaluated by physicians who are experts in the field of MG. Clinical subgroups were identified as EOMG or LOMG. MG disease severity was assessed through the Myasthenia Gravis Composite Scale (MGC) and MG Activities of Daily Living (MG-ADL) during blood collection (Tables 1 and S1).

#### Ethics statement

The study was approved by the Swedish Ethical Review Authority on human experimentation (ethical permit numbers 2020-03049 and 2023-02129-02) and the Riga Stradiņš University (ethical permit number Nr. 2-PĒK-4/70/2023), and written informed consent for research was obtained from all MG patients.

### Method details

#### Blood collection

Blood samples were collected in tubes without additives, stored at room temperature for at least 30-60 min, and then centrifuged at 2200 x g for 10 minutes. Serum samples were aliquoted, frozen, and stored at -80°C until further processing.

#### Proteomics analysis: proximity extension assay

Olink® Target 96 Inflammation panel (Olink Proteomics AB, Uppsala, Sweden), with Proximity Extension Assay (PEA) technology, analyzed 92 human proteins related to inflammation and various inflammatory diseases (Table S2). A total of 175 serum samples (from 98 AChR+ MG patients and 77 age- and sex-matched healthy controls) were randomly assigned across the two 96-well plates and subjected to the proteomic analyses performed at the Clinical Biomarkers Facility, Science for Life Laboratory, Uppsala University, Uppsala, Sweden without providing any demographic information about donors.

Briefly, PEA is based on antibody pairs equipped with DNA single-strand oligonucleotide reporter molecules, binding to their specific target protein. The DNA sequences are then extended and amplified by quantitative real-time PCR, and NPX values are calculated from the normalization of Ct values for individual samples and individual proteins.^26^^,^^27^ Four internal controls are added to each sample to monitor the quality of assay performance and individual samples. Samples that deviated less than 0.3 NPX from the median passed the quality control. Intra- and inter-coefficients of variance were based on internal controls in each plate. Statistical analysis was performed using either NPX (log scale values) or 2^NPX^ (linear scale values).

#### RNA isolation and qPCR

Serum samples were thawed on ice and centrifuged at 1500 x g for 5 min at 4°C. Total RNA, including miRNA, was isolated from 200 μl of serum with the miRNeasy® Serum/Plasma Advanced kit (QIAGEN®, Venlo, Netherlands). Synthetic RNA spike-in standards UniSp2, UniSp4, and UniSp5 (RNA Spike-in kit for RT, QIAGEN ®, Venlo, Netherlands) were added to the lysis buffer to monitor RNA isolation quality and efficiency. Additionally, 1 μg of MS2 bacteriophage RNA (Roche®, Basel, Switzerland) was added to the lysis buffer to increase the RNA yield. All RNA samples were eluted in 50 μl of nuclease-free water and stored at -80°C until further processing.

Isolated total RNA (2 μl) was used for cDNA synthesis in a 10 μl reverse transcription (RT) reaction using the miRCURY® LNA® RT kit (QIAGEN ®, Venlo, Netherlands) and an RNA Spike-in, UniSp6, was also added to control the RT efficiency and further monitoring in qPCR reaction as an interplate control. All cDNAs were synthesized and used directly or stored at -20°C or -80°C for up to 24 hours before performing real-time quantitative PCR.

Custom RT-qPCR 384 well-plate panels (miRCURY® LNA® miRNA custom PCR Panel, QIAGEN®, Venlo, Netherlands) were designed to contain primers for expression analysis for selected target (miR-30e-5p, miR-150-5p, and miR-21-5p) and endogenous miRNAs (miR-191-3p and miR-103a-3p), primers for hemolysis controls (miR-23a-3p and miR-451a), and primers for synthetic RNA Spike-in standards for quality control (UniSp2, UniSp4, UniSp5) and final calibration with interplate control (UniSp6). Using the ABI® QuantStudio® 6 Flex Real-Time PCR System (Thermo Fisher Scientific®, Waltham, Massachusetts, USA), a 10 μl RT-qPCR (containing 0.2 μl cDNA template) was performed using miRCURY® LNA® SYBR® Green PCR kit (QIAGEN®, Venlo, Netherlands) according to the manufactureŕs protocol in duplicate reactions.

Samples with a ΔCt of replicas > 1.5 or Ct >36 were excluded. A ΔCt of replicas > 1.5 was chosen as a cut-off to avoid the exclusion of samples with low miRNA concentration (high Ct), as increasing Ct variance is expected in these cases. Most replicas (∼96.5%) had a ΔCt < 0.5. Further quality control (QC) was evaluated by the expression of synthetic RNA spike-in standards and endogenous miRNAs suggested by the assay manufacturer (Qiagen®). An interplate calibration was obtained with the UniSp3. To exclude cellular miRNA contamination, all samples were checked for possible hemolysis using ΔCt (miR-23a-3p – miR-451a).

The miR-30e-5p, miR-150-5p, and miR-21-5p expression levels were determined by relative quantification, using the 2^(-ΔCt)^ method, where ΔCt = Mean Ct_(target miRNA)_ – Mean Ct_(endogenous miRNA)_. The miR-191-3p was used as a reference endogenous miRNA as it was most consistently expressed in this and previous studies.^6^^,^^7^^,^^28^

#### Protein and microRNA interactions

A miRNA target scan (https://www.targetscan.org/vert_80/) was performed to find binding sites of miR-150-5p, mir-30e-5p, and miR-21-5p on mRNAs encoding the significantly altered proteins.^29^ Since microRNAs bind to their mRNA specifically and reduce the protein translation and thereby, protein levels, relative values of miR-150-5p, miR-30e-5p, and miR-21-5p, respectively, were correlated with the 2ˆNPX values.

### Quantification and statistical analysis

Data were presented as normalized protein expression (NPX) values, OLINK Proteomics’ arbitrary unit on a log2 scale, and 2^NPX^, linear data. Each biomarker was analyzed against the binary variables using univariate and multiple logistic regression models i.e., Model 0, without, and Model 1, with adjustment for age and sex, respectively (Table S3). Odds ratios (OR) are presented per increase of one unit in each biomarker, which for a marker on the log2 scale indicates a doubling on the original scale. Statistical significance was defined with Bonferroni corrected P-values with the cut-off value at 0.05/11=0.00455 (for analysis of 11 replication biomarkers) or 0.05/92=0.000543 (for analysis of 92 biomarkers) for MG as an outcome and 0.05 for MG subgrouping as an outcome, as mentioned in figure legends. The C-statistic, presented for each protein marker, measures how well a marker discriminates between MG and HC, where 1 indicates perfect separation, and 0.5 indicates no separation or findings by chance.

Spearman correlation was performed to detect any correlation within the differentially expressed proteins and between differentially expressed proteins and miRNAs or demographic properties of patients and healthy individuals. Correlation plots are shown as a correlation matrix plotted with R-value with differing intensities indicating a degree of correlation. We only included correlations with p-values below 0.01, and correlations were classified as moderate (R = 0.40-0.69) and weak (R = 0.10-0.39) and positive or negative.^30^ Box-whisker plots are plotted as standard box plots with whiskers in the 10^th^-90^th^ percentile and individual data points. Standard PCA plots with PC1 and PC2 are plotted. Statistical analysis and graphs were made using GraphPad Prism (GraphPad Software Inc., San Diego, California, USA) and R version 4.2.3.

#### Random Forest and Boruta analysis

In addition to the logistic regression analyses, we applied the machine learning methods Random Forest and the Boruta algorithm. In contrast to the logistic regression models, the Random Forest models include all biomarkers simultaneously (i.e., they compete), and a non-linear association of biomarkers with the outcomes can be detected. Random Forest builds many decision trees, each on a bootstrapped dataset. In each branch, it chooses the square root of (92) biomarkers and finds the biomarker that makes the most difference in making the right decision about the outcome. When all trees are built, the difference between the correct decision for the original data is compared with the decision that would be obtained if the values of a biomarker are permuted. The difference is called “permutation importance” for that biomarker.

In Boruta, one starts with the Random Forest, where all biomarkers and their permuted copies (random noise) are included. This analysis compares which biomarkers are better than random noise. After each step, the importance of each biomarker is compared with the maximum importance from random noise. Biomarkers above random noise are confirmed, and those below random noise are rejected. The Random Forest was performed with biomarkers that remained until a decision was final for all biomarkers or until Random Forest had run 200 times. The biomarkers were sorted by the mean normalized permutation importance values, that is, the mean of the permutation importance calculated in each Random Forest divided by the standard deviation of the permutation importance. The Boruta algorithm was done with the R package Boruta.^31^
