# Supplementary material for: Serum protein biomarker profile distinguishes acetylcholine receptor antibody seropositive myasthenia gravis patients from healthy controls
Source: iScience. 2024 Jul 23;27(8):110564. doi: 10.1016/j.isci.2024.110564 (PMC11334828; doi:10.1016/j.isci.2024.110564)
Supplement: Document S1. Figure S1 and Tables S1–S4 [file mmc1.pdf]

iScience, Volume *27*

Supplemental information

Serum protein biomarker profile distinguishes acetylcholine receptor antibody seropositive myasthenia gravis patients from healthy controls

Amol K. Bhandage, Viktorija Keņina, Yu-Fang Huang, Marija Roddate, Gundega Kauke, Arta Grosmane, Violeta Žukova, Niclas Eriksson, Katja Gabrysch, Tanel Punga, and Anna Rostedt Punga

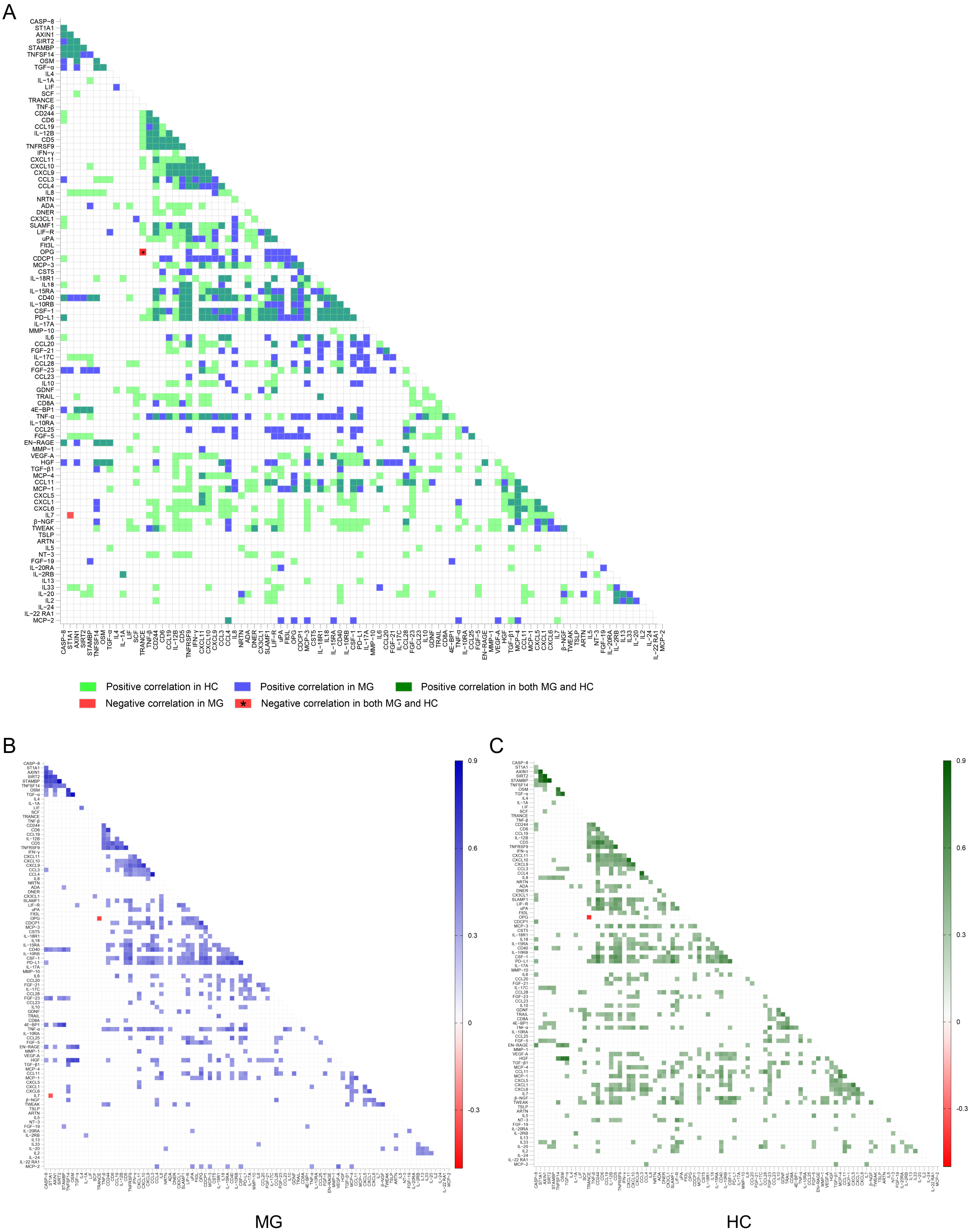

Figure S1. Correlation between the 92 inflammatory proteins. A, Matrix of the significant protein correlations in myasthenia gravis (MG) and healthy control (HC) groups. The correlation matrix for **B,** MG, and **C,** HC with color intensities indicative of R-value. Data are presented as Spearman R-values with significance level set at p ≤ 0.01.

Table S1. Detailed demographic characteristic of MG patients

#	Age/ sex	Disease duration (y)	Onset	IS	Medication	MG-ADL	MGC	Thymectomy (Tx)	Thymus
1	69/M	5	LOMG	No	AChEI	1	2	No	-
2	72/M	3	LOMG	No	None	1	2	No	-
3	78/M	7	LOMG	Yes	AChEI, Pred	5	9	No	-
4	67/F	9	LOMG	No	AChEI	6	12	No	-
5	76/F	7	LOMG	Yes	Aza	2	4	No	-
6	73/M	2	LOMG	No	AChEI	2	2	No	-
7	33/F	7	EOMG	Yes	AChEI, Aza	2	3		
8	67/M	2	LOMG	Yes	AChEi, Pred	0	0	No	-
9	34/F	3	EOMG	Yes	AChEI, Pred	9	16	No	-
10	74/M	24	LOMG	Yes	AChEI, Rtx	0	0	No	-
11	68/F	8	EOMG	No	None	2	4		
12	37/F	11	EOMG	No	None	0	0		
13	67/M	10	LOMG	Yes	Aza	0	0	No	-
14	76/F	7	LOMG	No	AChEI, Pred	5	9	No	-
15	59/F	16	EOMG	Yes	Pred, Aza	3	4	No	-
16	89/M	16	LOMG	Yes	AChEI, Pred	9	17	No	-
17	38/F	6	EOMG	Yes	AChEI, Pred, Tac, IvIG	12	21		
18	72/M	13	LOMG	Yes	Pred	1	1	No	-
19	19/F	3	EOMG	No	None	0	0	No	-
20	87/M	11	LOMG	Yes	Pred	0	0	No	
21	63/M	2	LOMG	Yes	AChEI	3	4	No	-
22	77/F	7	LOMG	Yes	AChEI, Aza	4	7	No	-
23	74/F	50	LOMG	No	AChEI	1	2	No	-
24	56/M	1	LOMG	No	AChEI	0	0	No	-
25	78/F	6	LOMG	Yes	Pred	4	8	No	-
26	73/M	4	LOMG	No	AChEI	0	0	No	-
27	22/F	0.5	EOMG	No	AChEI	8	11	Yes	Hpl
28	25/F	0.5	EOMG	No	AChEI	11	14	No	-
29	45/M	3	EOMG	Yes	AChEI, pred	12	14	Yes	Thymoma
30	41/F	11	EOMG	No	AChEI	0	0	Yes	Normal
31	38/F	14	EOMG	No	AChEI	0	0	Yes	-
32	56/M	29	EOMG	Yes	Rtx	8	11	Yes	Normal
33	28/F	1	EOMG	Yes	AChEI, pred	1	0	Yes	Hpl
34	30/F	14	EOMG	Yes	AChEI, pred, Aza, IvIG	1	1	Yes	Hpl
35	44/F	27	EOMG	No	AChEI	2	3	Yes	Hpl
36	31/M	0	EOMG	No	None	10	10	No	-
37	69/M	0	LOMG	No	AChEI	6	7	No	-
38	81/M	0	LOMG	No	None	16	17	No	-
39	66/F	2	LOMG	No	AChEI	14	13	No	-
40	56/F	26	EOMG	No	AChEI, pred	12	17	No	-
41	48/F	12	EOMG	Yes	AChEI, Aza	3	5	Yes	Normal
42	34/F	5	EOMG	Yes	AChEI, Rtx	3	5	Yes	Hpl
43	22/F	5.5	EOMG	Yes	AChEI, Pred, MMF	2	12	No	-
44	85/F	2	LOMG	Yes	AChEI, Pred, Mtx	0	5	No	-
45	36/F	7	EOMG	Yes	AChEI, Pred, Aza	3	6	Yes	Thymoma
46	56/M	7.5	EOMG	Yes	AChEI, Pred, MMF	6	7	No	-
47	58/F	0	LOMG	Yes	AChEI, Pred	7	14	No	-
48	64/M	0.5	LOMG	Yes	AChEI, MMF	8	13	No	-
49	56/M	2	LOMG	Yes	AChEI, Pred	3	5	No	-
50	48/M	8	EOMG	Yes	AChEI, Pred, Mtx	0	0	No	-
51	76/M	11	LOMG	Yes	AChEI, Pred, Aza	0	0	No	-
52	85/F	0.5	LOMG	No	AChEI	8	15	No	-
53	56/F	20.5	EOMG	Yes	Mtx	0	0	Yes	Thymoma
54	73/F	9.5	LOMG	Yes	Pred, Aza	0	0	No	-
55	38/M	10	EOMG	Yes	Pred, Aza	0	4	No	-
56	46/M	32	EOMG	No	AChEI	6	7	No	-

57	55/F	34	EOMG	Yes	AChEI, Pred, Aza	0	0	No	-
58	71/M	1.5	LOMG	Yes	AChEI, Pred, Aza	3	3	No	-
59	61/F	2	LOMG	Yes	AChEI, Aza	0	0	No	-
60	54/F	11.5	EOMG	Yes	AChEI, Pred, Mtx	0	0	No	-
61	60/M	0.5	LOMG	Yes	AChEI, Pred	1	1	No	-
62	75/F	8	LOMG	Yes	AChEI, Aza	5	5	No	-
63	45/F	7.5	EOMG	Yes	AChEI, MMF	3	n.a.	No	-
64	60/F	12	LOMG	Yes	Aza	1	1	Yes	Hpl
65	66/F	10	LOMG	Yes	AChEI, Pred	4	3	No	-
66	52/F	4	EOMG	Yes	AChEI, Pred, MMF	3	10	No	-
67	75/F	0.5	LOMG	Yes	AChEI, Aza	2	1	No	-
68	67/F	4.5	LOMG	Yes	AChEI, Mtx	1	2	Yes	Thymoma
69	44/F	5.5	EOMG	Yes	AChEI, Pred, Aza	4	10	Yes	Thymoma
70	62/M	1	LOMG	No	AChEI	0	0	Yes	Thymoma
71	61/F	13.5	EOMG	Yes	AChEI, Pred	0	5	Yes	Thymoma
72	51/M	2.5	EOMG	Yes	AChEI, Pred, Aza	5	7	No	-
73	65/M	2.5	LOMG	Yes	AChEI, Pred	2	4	No	-
74	54/M	4	EOMG	Yes	AChEI, Mtx	4	5	No	-
75	48/F	1.5	EOMG		AChEI, Pred, Aza	5	14	Yes	Thymoma
76	77/M	12.5	LOMG	Yes	AChEI, Pred, Aza	0	n.a.	No	-
77	38/F	13	EOMG	Yes	AChEI, Pred, MMF	9	21	No	-
78	64/M	3	LOMG	Yes	AChEI, Pred, Aza	1	2	No	-
79	81/F	2	LOMG	Yes	AChEI, Pred, MMF	0	0	No	-
80	47/F	7	EOMG	Yes	AChEI, Pred, Cya	0	0	Yes	Thymoma
81	40/M	8.5	EOMG	Yes	AChEI, Pred, Aza	0	0	No	-
82	86/F	3.5	LOMG	Yes	AChEI, Pred	0	0	No	-
83	62/M	1.5	LOMG	Yes	AChEI, Pred	0	0	No	-
84	64/F	46	EOMG	Yes	AChEI, Pred, Aza	7	13	Yes	Thymoma
85	77/F	3.5	LOMG	Yes	AChEI, Pred	7	14	No	-
86	59/F	11.5	EOMG	Yes	AChEI, Pred, Aza	4	8	No	-
87	61/F	4.5	LOMG	Yes	AChEI, Pred, Mtx	3	6	Yes	Thymoma
88	67/M	4	LOMG	Yes	AChEI, Pred, Mtx	0	0	No	-
89	51/F	3.5	EOMG	No	AChEI	0	0	No	-
90	29/F	4.5	EOMG	No	AChEI	0	0	No	-
91	34/M	5	EOMG	Yes	AChEI, Pred, Aza	6	14	Yes	Thymoma
92	43/F	12	EOMG	Yes	AChEI, Pred, Aza	3	9	Yes	Thymoma
93	37/F	0.5	EOMG	Yes	AChEI, Pred	1	2	Yes	Thymoma
94	39/F	27.5	EOMG	No	AChEI	6	11	No	-
95	77/M	3.5	LOMG	Yes	AChEI, Pred, Aza	0	0	No	-
96	66/F	7	LOMG	Yes	AChEI, Pred, Aza	0	4	No	-
97	43/F	0.5	EOMG	No	None	3	7	No	-
98	87/F	10	LOMG	No	None	1	6	No	-

LOMG, late-onset myasthenia gravis; EOMG, early-onset myasthenia gravis; IS, immunosuppression; AChEI, acetylcholinesterase inhibitors; Pred, prednisone; Aza, azathioprine; CyA, cyclosporine A; Rtx, Rituximab (every 6 months); Tac, tacrolimus; IvIG, intravenous immunoglobulins (every 4 weeks); Hpl, thymus hyperplasia; MMF, mycophenolate mofetil; Mtx, methotrexate; n.a., not available.

Table S2. Olink's Inflammation Panel, Protein assay list (Product number: 95302)

Adenosine Deaminase (ADA)	P00813	Fibroblast growth factor 23 (FGF-23)	Q9GZV9
Artemin (ARTN)	Q5T4W7	Fibroblast growth factor 5 (FGF-5)	Q8NF90
Axin-1 (AXIN1)	O15169	Fibroblast growth factor 19 (FGF-19)	O95750
Beta-nerve growth factor (β-NGF)	P01138	Fms-related tyrosine kinase 3 ligand (Flt3L)	P49771
Caspase-8 (CASP-8)	Q14790	Fractalkine (CX3CL1)	P78423
C-C motif chemokine 3 (CCL3)	P10147	Glial cell line-derived neurotrophic factor (GDNF)	P39905
C-C motif chemokine 4 (CCL4)	P13236	Hepatocyte growth factor (HGF)	P14210
C-C motif chemokine 19 (CCL19)	Q99731	Interferon gamma (IFN-γ)	P01579
C-C motif chemokine 20 (CCL20)	P78556	Interleukin-1 alpha (IL-1A)	P01583
C-C motif chemokine 23 (CCL23)	P55773	Interleukin-2 (IL-2)	P60568
C-C motif chemokine 25 (CCL25)	O15444	Interleukin-2 receptor subunit beta (IL-2RB)	P14784
C-C motif chemokine 28 (CCL28)	Q9NRJ3	Interleukin-4 (IL-4)	P05112
CD40L receptor (CD40)	P25942	Interleukin-5 (IL5)	P05113
CUB domain-containing protein 1 (CDCP1)	Q9H5V8	Interleukin-6 (IL6)	P05231
C-X-C motif chemokine 1 (CXCL1)	P09341	Interleukin-7 (IL-7)	P13232
C-X-C motif chemokine 5 (CXCL5)	P42830	Interleukin-8 (IL-8)	P10145
C-X-C motif chemokine 6 (CXCL6)	P80162	Interleukin-10 (IL10)	P22301
C-X-C motif chemokine 9 (CXCL9)	Q07325	Interleukin-10 receptor subunit alpha (IL-10RA)	Q13651
C-X-C motif chemokine 10 (CXCL10)	P02778	Interleukin-10 receptor subunit beta (IL-10RB)	Q08334
C-X-C motif chemokine 11 (CXCL11)	O14625	Interleukin-12 subunit beta (IL-12B)	P29460
Cystatin D (CST5)	P28325	Interleukin-13 (IL-13)	P35225
Delta and Notch-like epidermal growth factor-related receptor (DNER)	Q8NFT8	Interleukin-15 receptor subunit alpha (IL-15RA)	Q13261
Eotaxin (CCL11)	P51671	Interleukin-17A (IL-17A)	Q16552
Eukaryotic translation initiation factor 4E-binding protein 1 (4E-BP1)	Q13541	Interleukin-17C (IL-17C)	Q9P0M4
Fibroblast growth factor 21 (FGF-21)	Q9NSA1	Interleukin-18 (IL-18)	Q14116
Interleukin-18 receptor 1 (IL-18R1)	Q13478	Programmed cell death 1 ligand 1 (PD-L1)	Q9NZQ7
Interleukin-20 (IL-20)	Q9NYY1	Protein S100-A12 (EN-RAGE)	P80511
Interleukin-20 receptor subunit alpha (IL-20RA)	Q9UHF4	Signaling lymphocytic activation molecule (SLAMF1)	Q13291
Interleukin-22 receptor subunit alpha-1 (IL-22 RA1)	Q8N6P7	SIR2-like protein 2 (SIRT2)	Q8IXJ6
Interleukin-24 (IL-24)	Q13007	STAM-binding protein (STAMBP)	O95630
Interleukin-33 (IL-33)	O95760	Stem cell factor (SCF)	P21583
Latency-associated peptide transforming growth factor β1 (TGF-β1)	P01137	Sulfotransferase 1A1 (ST1A1)	P50225
Leukemia inhibitory factor (LIF)	P15018	T cell surface glycoprotein CD6 isoform (CD6)	Q8WWJ7
Leukemia inhibitory factor receptor (LIF-R)	P42702	T-cell surface glycoprotein CD5 (CD5)	P06127
Macrophage colony-stimulating factor 1 (CSF-1)	P09603	T-cell surface glycoprotein CD8 alpha chain (CD8A)	P01732
Matrix metalloproteinase-1 (MMP-1)	P03956	Thymic stromal lymphopoietin (TSLP)	Q969D9
Matrix metalloproteinase-10 (MMP-10)	P09238	TNF-beta (TNF-β)	P01374
Monocyte chemotactic protein 1 (MCP-1)	P13500	TNF-related activation-induced cytokine (TRANCE)	O14788
Monocyte chemotactic protein 2 (MCP-2)	P80075	TNF-related apoptosis-inducing ligand (TRAIL)	P50591
Monocyte chemotactic protein 3 (MCP-3)	P80098	Transforming growth factor alpha (TGF-α)	P01135
Monocyte chemotactic protein 4 (MCP-4)	Q99616	Tumor necrosis factor (Ligand) superfamily, member 12 (TWEAK)	O43508
Natural killer cell receptor 2B4 (CD244)	Q9BZW8	Tumor necrosis factor alpha (TNF-α)	P01375
Neurotrophin-3 (NT-3)	P20783	Tumor necrosis factor ligand superfamily member 14 (TNFSF14)	O43557
Neurturin (NRTN)	Q99748	Tumor necrosis factor receptor superfamily member 9 (TNFRSF9)	Q07011
Oncostatin-M (OSM)	P13725	Urokinase-type plasminogen activator (uPA)	P00749
Osteoprotegerin (OPG)	O00300	Vascular endothelial growth factor A (VEGF-A)	P15692

Table S3. Univariate and bivariate logistic regression analysis of 92 inflammatory proteins, with MG as an outcome (reference group), using Model 0 (not corrected for age and sex) and Model 1 (corrected for age and sex), respectively.

Proteins	Model 0				Model 1			
	OR(95% CI)	P-value	Sign.	C-stat	OR(95% CI)	P-value	Sign.	C-stat
ST1A1	0.33(0.24-0.45)	5.30E-12	***	0.873	0.33(0.24-0.46)	1.10E-11	***	0.876
TNFSF14	0.08(0.04-0.17)	2.20E-11	***	0.897	0.08(0.04-0.17)	3.40E-11	***	0.902
TGF-α	0.07(0.03-0.16)	8.80E-11	***	0.869	0.07(0.03-0.16)	1.90E-10	***	0.879
CCL28	48.33(14.71-158.78)	1.70E-10	***	0.890	52.84(15.33-182.19)	3.30E-10	***	0.893
4E-BP1	4.71(2.83-7.82)	2.30E-09	***	0.871	4.97(2.90-8.52)	5.10E-09	***	0.884
FGF-23	18.53(7.06-48.59)	3.00E-09	***	0.825	22.07(7.63-63.81)	1.10E-08	***	0.836
IL7	4.45(2.50-7.91)	3.60E-07	***	0.750	4.69(2.60-8.46)	3.00E-07	***	0.768
CCL20	3.28(2.05-5.24)	7.00E-07	***	0.759	3.27(2.03-5.27)	1.10E-06	***	0.764
IL10	5.14(2.66-9.94)	1.10E-06	***	0.782	5.18(2.64-10.18)	1.80E-06	***	0.792
IL6	3.37(2.08-5.45)	7.80E-07	***	0.769	3.22(1.98-5.23)	2.20E-06	***	0.770
OSM	0.34(0.22-0.53)	2.00E-06	***	0.724	0.34(0.22-0.54)	4.00E-06	***	0.740
IL-17C	3.51(2.05-6.00)	4.70E-06	***	0.711	3.70(2.10-6.51)	5.50E-06	***	0.735
FGF-5	17.04(5.04-57.62)	5.10E-06	***	0.716	15.46(4.41-54.23)	1.90E-05	***	0.731
GDNF	4.96(2.38-10.34)	1.90E-05	***	0.708	5.09(2.41-10.74)	1.90E-05	***	0.730
SIRT2	0.46(0.32-0.65)	9.60E-06	***	0.764	0.47(0.33-0.67)	2.70E-05	***	0.771
FGF-21	1.74(1.35-2.23)	1.40E-05	***	0.705	1.70(1.32-2.20)	3.90E-05	***	0.729
VEGFA	3.62(2.02-6.48)	1.50E-05	***	0.707	3.37(1.87-6.06)	5.20E-05	***	0.730
TNF	4.78(2.26-10.08)	4.10E-05	***	0.690	4.56(2.17-9.62)	6.50E-05	***	0.706
CCL11	5.00(2.33-10.72)	3.70E-05	***	0.696	4.64(2.10-10.25)	0.0002	***	0.711
CD6	0.34(0.18-0.64)	0.0008	***	0.655	0.30(0.16-0.58)	0.0003	***	0.693
MCP-3	2.95(1.73-5.02)	6.70E-05	***	0.693	2.73(1.59-4.68)	0.0003	***	0.700
uPA	0.12(0.04-0.40)	0.0005	***	0.670	0.10(0.03-0.35)	0.0003	***	0.720
EN-RAGE	2.01(1.35-2.99)	0.0006	***	0.681	2.08(1.37-3.16)	0.0005	***	0.714
CDCP1	2.92(1.68-5.08)	0.0002	***	0.680	2.67(1.52-4.68)	0.0006	***	0.682
IL4	0.43(0.27-0.69)	0.0005	***	0.651	0.43(0.26-0.71)	0.0008	***	0.685
TGF-β1	2.73(1.40-5.35)	0.0033	**	0.648	3.26(1.61-6.60)	0.0010	***	0.701
TNF-β	0.30(0.15-0.58)	0.0004	***	0.668	0.30(0.15-0.62)	0.0010	***	0.705
HGF	3.43(1.70-6.94)	0.0006	***	0.663	3.27(1.60-6.68)	0.0012	**	0.685
CXCL10	1.97(1.32-2.95)	0.0009	***	0.662	1.93(1.29-2.90)	0.0015	**	0.679
CXCL11	1.91(1.28-2.84)	0.0016	**	0.660	1.92(1.28-2.89)	0.0017	**	0.668
AXIN1	0.48(0.32-0.73)	0.0006	***	0.709	0.51(0.33-0.78)	0.0018	**	0.720
MMP-10	2.44(1.35-4.42)	0.0030	**	0.635	2.45(1.35-4.45)	0.0031	**	0.667
CXCL9	2.05(1.35-3.12)	0.0008	***	0.665	1.88(1.23-2.90)	0.0039	**	0.655
STAMBP	0.40(0.22-0.73)	0.0027	**	0.723	0.42(0.22-0.77)	0.0053	**	0.738
IL8	0.60(0.40-0.89)	0.0116	*	0.605	0.55(0.36-0.84)	0.0054	**	0.671
MCP-1	2.92(1.44-5.95)	0.0031	**	0.616	2.69(1.31-5.55)	0.0071	**	0.653
CD244	0.24(0.09-0.62)	0.0033	**	0.617	0.26(0.10-0.70)	0.0075	**	0.653
MMP-1	1.83(1.19-2.80)	0.0059	**	0.636	1.75(1.13-2.71)	0.0114	*	0.658
IL-17A	2.35(1.25-4.44)	0.0083	**	0.613	2.27(1.19-4.35)	0.0132	*	0.636
CXCL5	1.77(1.16-2.72)	0.0082	**	0.638	1.74(1.11-2.73)	0.0149	*	0.654
CD8A	1.48(0.99-2.21)	0.0570		0.581	1.61(1.05-2.47)	0.0284	*	0.653
IL18	1.99(1.10-3.63)	0.0239	*	0.590	1.99(1.07-3.70)	0.0293	*	0.638
Flt3L	2.17(1.16-4.08)	0.0155	*	0.612	1.98(1.04-3.75)	0.0374	*	0.645
CST5	1.91(1.07-3.39)	0.0287	*	0.578	1.85(1.02-3.33)	0.0414	*	0.630
MCP-4	1.80(1.10-2.93)	0.0182	*	0.606	1.67(1.02-2.73)	0.0419	*	0.626
ADA	0.45(0.22-0.92)	0.0278	*	0.611	0.48(0.23-0.99)	0.0456	*	0.630
PD-L1	2.14(0.96-4.73)	0.0617		0.567	2.24(0.99-5.07)	0.0535		0.634
CX3CL1	0.61(0.33-1.13)	0.1136		0.556	0.53(0.28-1.02)	0.0571		0.635
DNER	0.28(0.09-0.87)	0.0273	*	0.583	0.33(0.10-1.04)	0.0589		0.633
IL-1A	1.86(0.86-4.03)	0.1138		0.557	2.12(0.95-4.71)	0.0661		0.633
IFN-γ	1.32(0.98-1.77)	0.0696		0.596	1.32(0.98-1.79)	0.0684		0.626
IL13	0.68(0.45-1.05)	0.0812		0.524	0.67(0.43-1.05)	0.0807		0.620
CXCL1	1.61(0.93-2.80)	0.0909		0.582	1.68(0.94-3.00)	0.0810		0.636
CCL23	1.74(0.89-3.39)	0.1075		0.578	1.78(0.90-3.51)	0.0970		0.628

TRAIL	1.76(0.76-4.11)	0.1883	0.544	1.97(0.82-4.75)	0.1285	0.629
IL-18R1	1.72(0.87-3.40)	0.1188	0.559	1.71(0.85-3.44)	0.1343	0.631
IL-10RB	2.69(0.91-7.97)	0.0733	0.579	2.32(0.76-7.06)	0.1395	0.621
CASP-8	0.71(0.49-1.04)	0.0823	0.644	0.75(0.51-1.10)	0.1428	0.654
CCL25	1.62(0.96-2.75)	0.0723	0.572	1.49(0.87-2.56)	0.1464	0.623
LIF-R	2.22(0.74-6.62)	0.1529	0.606	2.18(0.73-6.53)	0.1644	0.623
CCL3	0.78(0.50-1.21)	0.2691	0.569	0.73(0.46-1.15)	0.1722	0.630
CD5	0.66(0.31-1.39)	0.2741	0.551	0.60(0.28-1.30)	0.1983	0.624
CD40	0.66(0.31-1.41)	0.2840	0.587	0.61(0.28-1.33)	0.2122	0.619
IL-20RA	1.40(0.76-2.56)	0.2784	0.547	1.47(0.80-2.70)	0.2146	0.623
IL2	2.04(0.70-5.93)	0.1902	0.563	1.95(0.65-5.83)	0.2326	0.622
CCL19	1.22(0.86-1.72)	0.2607	0.544	1.23(0.87-1.75)	0.2448	0.620
IL-24	1.46(0.78-2.74)	0.2410	0.542	1.45(0.77-2.75)	0.2535	0.619
SLAMF1	0.76(0.40-1.44)	0.3963	0.519	0.70(0.36-1.36)	0.2887	0.611
LIF	1.46(0.66-3.25)	0.3533	0.511	1.48(0.66-3.29)	0.3400	0.613
IL33	1.64(0.57-4.66)	0.3568	0.616	1.63(0.56-4.73)	0.3650	0.625
MCP-2	1.29(0.83-2.00)	0.2568	0.538	1.22(0.78-1.91)	0.3879	0.617
IL-15RA	1.93(0.67-5.62)	0.2257	0.531	1.60(0.53-4.82)	0.4040	0.608
CSF-1	1.81(0.51-6.48)	0.3616	0.564	1.61(0.44-5.93)	0.4738	0.614
SCF	1.20(0.59-2.44)	0.6104	0.562	1.30(0.63-2.66)	0.4813	0.603
FGF-19	1.17(0.87-1.55)	0.2975	0.547	1.11(0.82-1.50)	0.4839	0.610
IL-20	1.33(0.51-3.50)	0.5576	0.612	1.40(0.53-3.70)	0.5035	0.609
ARTN	0.82(0.48-1.42)	0.4798	0.531	0.83(0.47-1.46)	0.5249	0.609
IL-10RA	1.19(0.85-1.66)	0.3085	0.553	1.12(0.79-1.58)	0.5277	0.613
CXCL6	1.16(0.74-1.80)	0.5218	0.522	1.15(0.73-1.81)	0.5420	0.610
TWEAK	1.11(0.49-2.52)	0.8067	0.519	1.27(0.54-2.99)	0.5818	0.608
IL-2RB	1.04(0.61-1.77)	0.8830	0.503	1.12(0.65-1.95)	0.6812	0.609
TRANCE	0.94(0.62-1.41)	0.7571	0.501	1.07(0.70-1.66)	0.7442	0.608
β-NGF	1.67(0.07-39.80)	0.7512	0.513	1.69(0.07-42.82)	0.7515	0.609
CCL4	0.98(0.63-1.53)	0.9365	0.504	0.93(0.59-1.47)	0.7652	0.614
TSLP	0.96(0.63-1.45)	0.8355	0.505	0.94(0.61-1.44)	0.7653	0.608
IL-12B	0.95(0.67-1.36)	0.7886	0.509	0.95(0.67-1.37)	0.8026	0.608
NT-3	0.86(0.52-1.42)	0.5540	0.501	0.95(0.57-1.60)	0.8569	0.609
NRTN	1.05(0.56-1.97)	0.8864	0.540	1.06(0.56-2.00)	0.8637	0.610
TNFRSF9	0.99(0.60-1.62)	0.9562	0.506	0.96(0.58-1.60)	0.8822	0.608
IL5	0.91(0.64-1.30)	0.6042	0.553	0.98(0.67-1.42)	0.9064	0.611
IL-22 RA1	1.04(0.69-1.59)	0.8370	0.502	1.01(0.66-1.56)	0.9525	0.610
OPG	1.37(0.65-2.88)	0.4132	0.521	1.00(0.45-2.21)	0.9915	0.610

OR, odds ratio; CI, confidence interval. Column “sign.” represents * p<0.05, ** p<0.01, *** p<0.001.

Table S4. Univariate logistic regression analysis of inflammatory proteins with LOMG, immunosuppression, disease severity, thymectomy, and thymoma as outcome (reference group) within subgroups of MG patients (related to Fig 4).

Proteins	OR	(95 % CI)	P-value	Sign.	C-stat
EOMG vs **LOMG**					
TGF-β1	0.288	(0.1012 - 0.7241)	0.0124	*	0.664
OPG	2.506	(1.033 - 6.501)	0.0483	*	0.631
Immunosuppression (IS- vs **IS+**)					
CXCL10	0.467	(0.270 - 0.753)	0.0032	**	0.703
CCL11	4.354	(1.564 - 13.66)	0.0071	**	0.681
IL-17C	2.113	(1.116 - 4.471)	0.0329	*	0.626
TGF-α	2.665	(1.281 - 5.997)	0.012	*	0.673
TNFSF14	2.587	(1.410 - 5.110)	0.0035	**	0.706
MG-ADL 0-4 vs **≥5**					
uPA	0.068	(0.010 - 0.363)	0.0033	**	0.698
Thymectomy (-Tx- vs **+Tx**)					
OPG	0.146	(0.037 - 0.474)	0.0029	**	0.715
CCL20	0.262	(0.104 - 0.554)	0.0016	**	0.734
**Thymoma** vs non-thymoma					
IL-22 RA1	3.38	(1.572 - 8.714)	0.0047	**	0.719
uPA	0.06	(0.006 - 0.424)	0.0074	**	0.78

OR, odds ratio; CI, confidence interval. Column “sign.” represents * $p<0.05$, ** $p<0.01$.
The subgroup name in bold and underlined text indicates “outcome” or “reference” group in the logistic regression analysis.
